# Crystallization Features of Amorphous Rapidly Quenched High Cu Content TiNiCu Alloys upon Severe Plastic Deformation

**DOI:** 10.3390/ma12172670

**Published:** 2019-08-22

**Authors:** Alexander Glezer, Nikolay Sitnikov, Roman Sundeev, Alexander Shelyakov, Irina Khabibullina

**Affiliations:** 1 National University of Science and Technology “MISIS”, Leninskii pr. 4, 119049 Moscow, Russia; 2 Federal State Unitary Enterprise “Keldysh Research Center”, Onezhskaya st. 8, 125438 Moscow, Russia; 3 National Research Nuclear University MEPhI (Moscow Engineering Physics Institute), Kashirskoe shosse 31, 115409 Moscow, Russia; 4 “MIREA–Russian Technological University”, Vernadskogo pr. 78, 119454 Moscow, Russia

**Keywords:** amorphous state, melt spinning, severe plastic deformation, high-pressure torsion, microstructure, crystallization, phase transformation

## Abstract

In recent years, the methods of severe plastic deformation and rapid melt quenching have proven to be an effective tool for the formation of the unique properties of materials. The effect of high-pressure torsion (HPT) on the structure of the amorphous alloys of the quasi-binary TiNi–TiCu system with a copper content of more than 30 at.% produced by melt spinning technique has been analyzed using the methods of scanning electron microscopy, X-ray diffraction analysis, and differential scanning calorimetry (DSC). The structure examinations have shown that the HPT of the alloys with a Cu content ranging from 30 to 40 at.% leads to nanocrystallization from the amorphous state. An increase in the degree of deformation leads to a substantial change in the character of the crystallization reflected by the DSC curves of the alloys under study. The alloys containing less than 34 at.% Cu exhibit crystallization peak splitting, whereas the alloys containing more than 34 at.% Cu exhibit a third peak at lower temperatures. The latter effect suggests the formation of regions of possible low-temperature crystallization. It has been established that the HPT causes a significant decrease in the thermal effect of crystallization upon heating of the alloys with a high copper content relative to that of the initial amorphous melt quenched state.

## 1. Introduction

In accordance with modern trends in the development of science and technology, advanced industries urgently require “smart” multifunctional materials combining high-performance characteristics in addition to unique properties. Alloys with shape memory effect (SME) are a prime example of such a material. In recent years, the efficiency and prospects of the use of SME alloys in various fields of technology have been demonstrated, in particular, in aerospace engineering, medicine, and robotics [1,2,3,4]. The design of high-speed (especially fast cyclic response) devices requires thin SME materials (thin ribbons, wires, or films) characterized by narrow hysteresis of martensitic phase transformations. The materials meeting such requirements include the alloys of the quasi-binary intermetallic TiNi–TiCu system with a copper content of more than 10 at.% [5]. However, the TiNiCu alloys with high copper content are brittle because of the TiCu phase formation near grain boundaries and, therefore, cannot be deformed into wire or ribbon upon hot or cold processing [6,7], which is necessary for the manufacture of thermal actuators. One of the best alternatives for overcoming the brittleness of the alloys is the method of rapid melt quenching [8,9,10]. Such extreme action allows one to produce thin ribbons directly from the molten metal, and a high solidification rate suppresses the formation of TiCu particles. The specific feature of the rapidly quenched TiNiCu alloys with high copper content is that they can be obtained in the amorphous state at high cooling rates, and can later be transformed to a crystalline state with a pronounced SME [8,9,10] by means of a variety of external actions, including isothermal annealing, laser, and electropulse treatment.

In recent years, the physical materials science has been enriched by a new scientific direction in which the production of structural and functional bulk nanostructured materials is provided by the methods of severe plastic deformation (SPD). It is known that the SPD methods allow one to obtain nano- and submicrocrystalline materials with special mechanical characteristics substantially differing from the properties typical of conventional polycrystalline materials. The highest degrees of deformation are achieved by shear plastic deformation in laboratory conditions on experimental samples of various metals and alloys by high-pressure torsion (HPT) in the Bridgman anvils. Recently, we have shown that HPT of the initially amorphous or initially crystalline TiNi–TiCu alloy with 25 at.% copper leads to the realization of several cycles of interrelated phase transformations of the “crystal-amorphous state” type [11]. This phenomenon can be explained by the model of the superposition of various mechanical energy dissipation channels upon SPD. It was also shown that the tendencies to amorphization upon rapid melt quenching and HPT for the same alloy substantially differ, and the “amorphous state-crystal” reverse phase transformation is almost always accompanied by the formation of a nanocrystalline structure [12]. The aim of this work was to study the effect of SPD by torsion under high quasi-hydrostatic pressure on the structure formation in the TiNi–TiCu alloys with a copper content of more than 30 at.%.

## 2. Materials and Methods 

**Figure 1:**
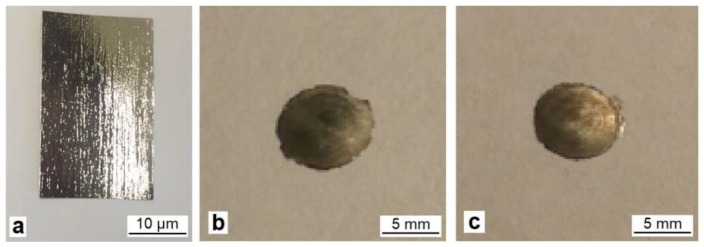
TiNiCu alloy samples in the initial state (**a**) and after deformation to *n* = 3 (**b**) and *n* = 5 (**c**).

The alloys of the quasi-binary TiNi–TiCu system with a constant titanium content of 50 at.% and a copper content ranging from 30 to 40 at.% have been obtained by melt quenching [8]. The initial ingots of the alloys of required compositions were prepared from high-purity metals (H0 grade electrode nickel, M0 grade oxygen-free copper, and iodide titanium) with sixfold remelting in an arc furnace in an argon atmosphere to ensure homogeneity. The obtained ingots were melted in a quartz crucible in a helium atmosphere and extruded through a narrow nozzle in the crucible onto the surface of a rapidly rotating copper wheel. The process occurring at a melt cooling rate of about 10^6^ K/s resulted in the manufacture of ribbons 30–50 μm thick and 8–20 mm wide (Figure 1a).

Upon the HPT deformation method used in the work, the sample is placed between two anvils and compressed under a quasi-hydrostatic pressure of several GPa [13,14]. The lower anvil rotates, and the sample is deformed by shear under the action of surface friction. Since deformation occurs under conditions of quasi-hydrostatic compression, the sample does not fail and is subjected to severe deformation.

The TiNiCu alloy samples were deformed by HPT in a Bridgman cell at room temperature at a quasi-hydrostatic pressure of 6 GPa and at a rotation speed of the movable anvil of 1 rpm to the degree of deformation corresponding to *n* = 1, 3, and 5, where *n* is the number of complete revolutions of the movable anvil. The samples for HPT deformation were folded into a packet of 3 layers and fastened at several points by microwelding. Such deformation resulted in the preparation of disk shape samples of 8 mm in diameter (Figure 1b,c).

The structure of the alloys was studied by metallography, optical and electron microscopy, and X-ray diffraction (XRD). For metallographic studies of the ribbon samples, their transverse polished sections were prepared with Buehler equipment (Lake Bluff, IL, USA). The polished surface was etched with an HF (5%) + H_2_SO_4_ (5%) + H_2_NO_3_ (25%) + H_2_O (70%) solution. The microstructure of the surfaces and cross sections of the samples was studied with a Carl Zeiss Axiovert 40 MAT inverted metallographic microscope (Oberkochen, Germany) with a reflected light and an FEI Quanta 600 FEG scanning electron microscope (Hillsboro, OR, USA) with an X-ray electron-probe microanalyzer (EDAX). The XRD analysis was performed with a PANalytical Empyrean diffractometer (Almelo, The Netherland) by Bragg–Brentano focusing using a hybrid monochromator in СuК_α_ radiation and a DRON-3М diffractometer (Burevestnik, Russia) in CoK_α_ radiation (in an angle range of 10–120° at a step of 0.1°; exposure time was 5 s) at room temperature.

For the determination of the devitrification temperature and for the analysis of thermal crystallization processes, the rapidly quenched alloys were subjected to controlled annealing in an NETZSCH STA 449 F1 Jupiter differential scanning calorimeter (Selb, Germany) at a heating rate of 10 K/min.

## 3. Results

### 3.1. Melt Quenching

**Figure 2:**
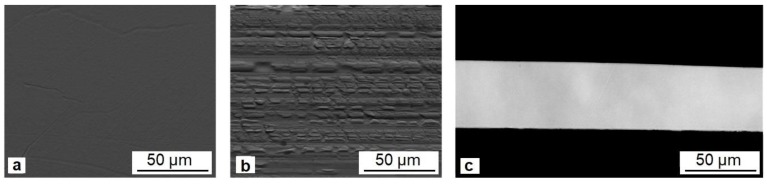
Typical images of non-contact (**a**) and contact (**b**) surfaces and cross section (**c**) of rapidly quenched TiNiCu ribbons (for the alloy with 34 at.% Cu).

Typical images of the surfaces of rapidly quenched ribbons are shown in Figure 2. The non-contact side of the ribbons (Figure 2a) is characterized by continuous uniform surface, which is almost free from defects, and the contact side of the ribbons (Figure 2b) exhibits a relief replicated from the quenching wheel.

The cross-section samples of all melt spun ribbons exhibit homogeneous amorphous structure. A typical cross-section ribbon structure is shown in Figure 2c for the ribbon containing 34 at.% copper.

**Figure 3:**
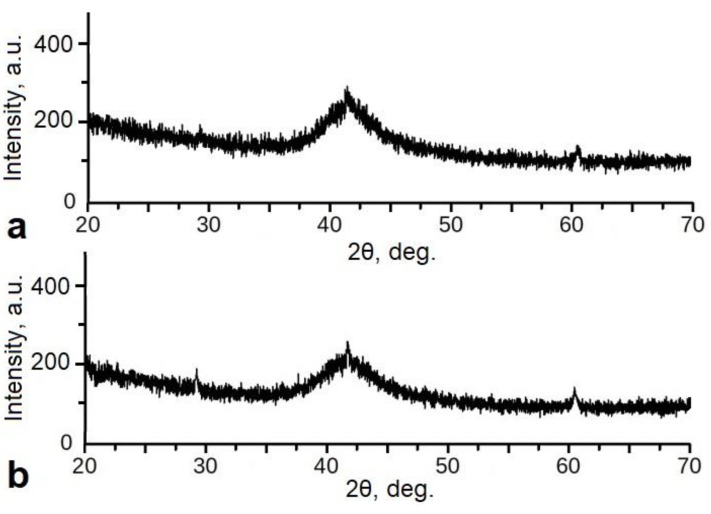
Typical XRD patterns of the as-quenched TiNiCu alloy ribbons with 30 at.% Cu from the contact (**a**) and non-contact (**b**) sides.

The XRD patterns of the non-contact (free) and contact surfaces of all as-quenched TiNiCu alloy ribbons (Figure 3) exhibit a diffuse amorphous halo at 2θ = 40–45° (СuК_α_ radiation), which indicates the amorphous state of the alloys. However, small peaks from crystalline phases are observed in the XRD pattern of the sample with 30 at.% Cu, which demonstrates the presence of a small fraction of crystallites in the amorphous matrix.

### 3.2. High-Pressure Torsion

**Figure 4:**
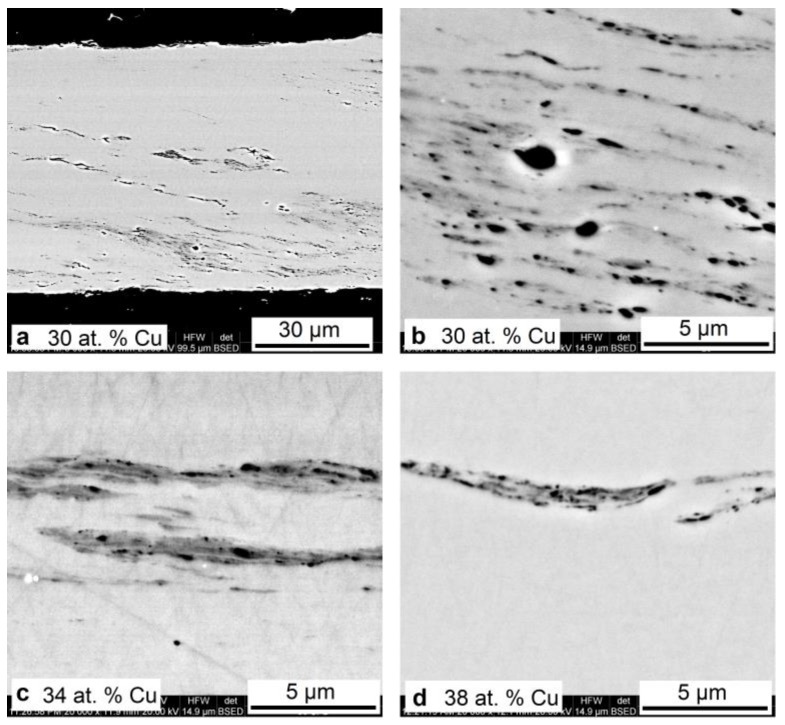
Cross section of the melt spun TiNiCu ribbons containing 30 at.% Cu (**a,b**), 34 at.% Cu (**c**), and 38 at.% Cu (**d**) after high-pressure torsion (HPT) to *n* = 5.

The cross-sectional microstructure of the ribbons subjected to HPT contains elongated structure elements which may be caused by shear deformation of the amorphous matrix and, in particular, could represent crystalline nanoparticles resulting from partial crystallization of the amorphous phase (Figure 4). Shear deformation regions with the stratification of the structure are observed mainly near the surfaces of the alloys and are characterized by submicro- and nanodimensions and porosity. At the same time, the number of such structure elements is highest in the alloy with 30 at.% copper and noticeably decreases with increasing copper content in the alloys. This is apparently due to a greater degree of amorphization of the alloys with a copper content of more than 30 at.%.

**Figure 5:**
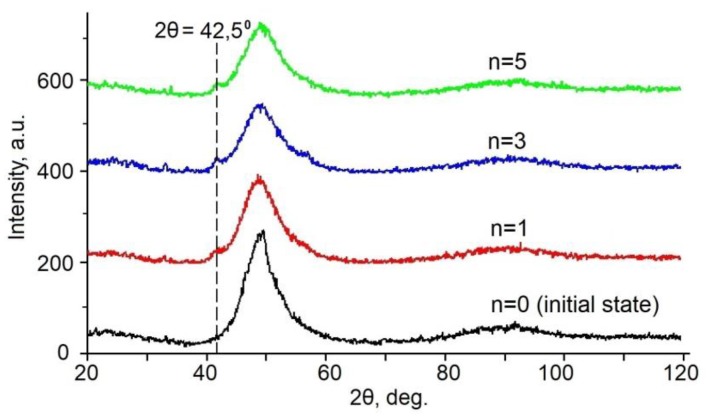
XRD patterns of the TiNiCu alloy with 34 at.% Cu in the initial state and after HPT to *n* = 1, 3, and 5.

The XRD examination revealed changes in the structure of the alloys after HPT to *n* ≥ 1 (Figure 5). It is seen that the HPT of the alloy is accompanied by the appearance of the diffraction line belonging to the crystalline phase in the XRD spectra at 2θ = 42.5° against the background of the amorphous halo (CoK_α_ radiation). Unfortunately, we failed to unambiguously identify the crystalline phase because of a low intensity and a small number of the diffraction lines.

### 3.3. Crystallization in Calorimeter (DSC)

**Figure 6:**
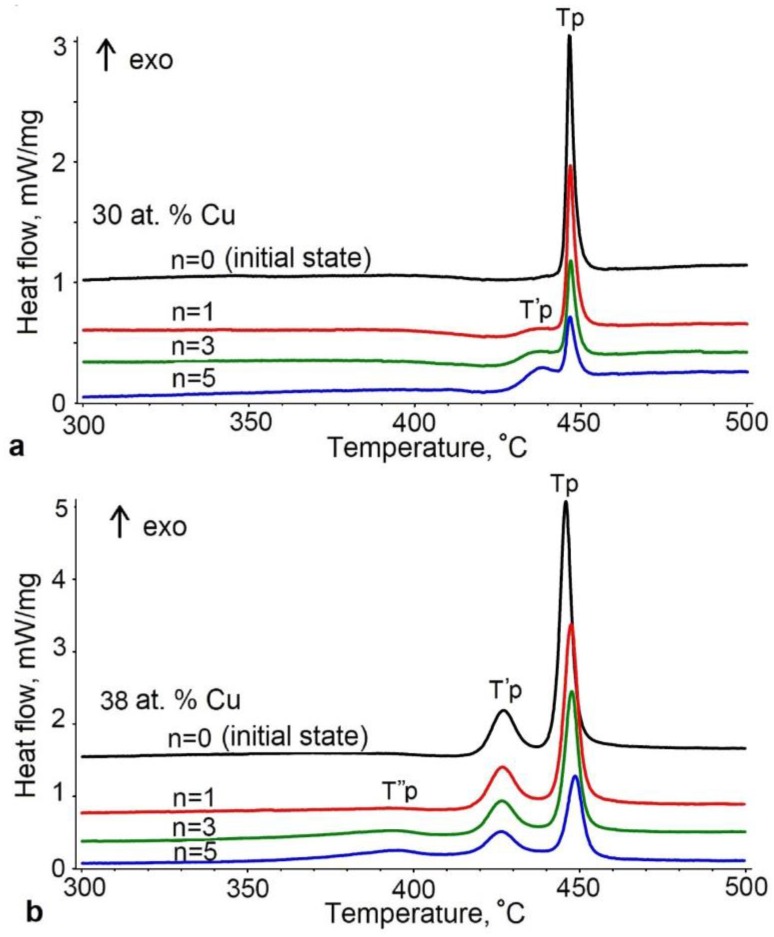
DSC curves for the crystallization of the TiNiCu alloys with 30 at.% Cu (**a**) and 38 at.% Cu (**b**) in the initial state and after HPT to *n* = 1, 3, and 5.

Typical DSC curves of the crystallization of rapidly quenched ribbons in the calorimeter are shown in Figure 6. The curves of the as-quenched alloys with a copper content of up to 34 at.% exhibit one peak of heat release (at crystallization peak temperature T_p_), which indicates a single-stage crystallization, while the curves of the ribbons with a copper content of more than 34 at.% exhibit two separate heat release peaks, which are responsible for the two-stage thermal crystallization. In this case, the low-temperature peak (at crystallization peak temperature T_p_) is close in the magnitude of the energy release to the crystallization enthalpy of the samples with copper contents below 34 at.%, while the high-temperature peak is higher by a factor of about 3. Therefore, we can conclude that the crystallization peak at higher temperature is related to the formation of the phases differing from those formed in the samples with copper contents below 34 at.%. Note that the T_p_ temperature does not virtually change with increasing copper content.

An increase in the degree of deformation leads to a noticeable change in the character of crystallization in the DSC curves. First, there is a split of peaks for the alloys with Cu content below 34 at.% and the appearance of a third peak at lower temperatures (crystallization peak temperature *T^″^_p_*) for the alloys with more than 34 at.% Cu. This suggests that HPT of the alloys results in the formation of regions in which crystallization is possible at lower temperatures.

Second, the results of DSC (Table 1) unambiguously indicate that HPT of amorphous melt-spun TiNiCu alloys leads to a significant change in the thermal effect of crystallization upon continuous heating of the alloys. After HPT, the energy consumption of the crystallization of the TiNiCu alloys decreases relative to that of the same material upon annealing without deformation. 

**Figure 7:**
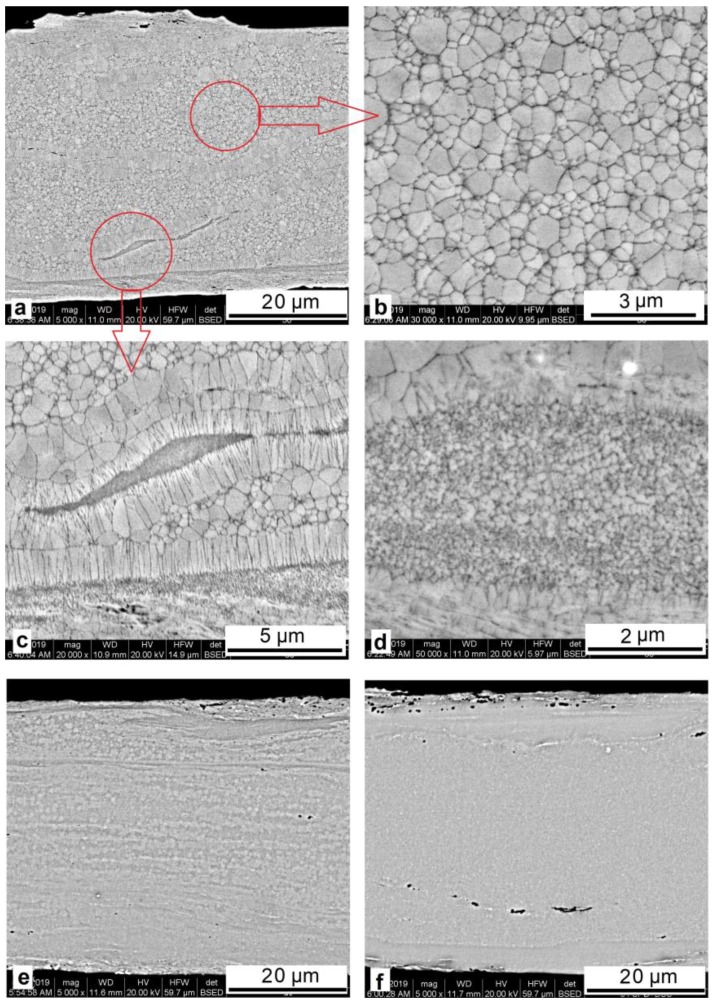
Cross section of the melt-spun TiNiCu ribbons containing 30 at.% Cu (**a**–**d**), 34 at.% Cu (**e**), and 38 at.% Cu (**f**) after HPT (*n* = 5) and crystallization in the calorimeter.

The structure formed in the alloys upon crystallization in the DSC device is characterized by inhomogeneity in the cross section of the sample with stratification by structure elements (Figure 7). The alloy with a copper content of 30 at.% predominantly exhibits the regions of the B19 martensitic structure and an average grain size of 0.5–0.7 μm (Figure 7b) typical of the alloys after isothermal crystallization [8].

The finer (nanosized) subsurface structure formed upon shear deformation of the amorphous phase is bounded by extended columnar crystals formed across the deformation axis upon HPT (Figure 7c,d). A slightly different picture observed in the alloys with a copper content of 34 at.% and higher (Figure 7e,f) is most likely due to the predominant formation of Ti–Cu phases in the ribbons [15].

## 4. Discussion

As noted above, rapidly quenched alloys of the TiNi–TiCu intermetallic system have attracted substantial interest from researchers in the context of their unusual structure characteristics and phase transformations and, from a practical point of view, as an advanced shape memory material for micromechanical applications. Recently, a number of papers have appeared on the properties of melt-spun TiNiCu alloys subjected to SPD [16,17,18,19,20]. However, most the works used an alloy with 25 at.% Cu primarily due to its high tendency to amorphization and, therefore, the ability to obtain it in amorphous state at cooling rates is achievable by melt spinning technology (about 10^6^ К/s). It was found that the combined effect of rapid solidification SPD by HPT and subsequent annealing can lead to the formation of homogeneous nanostructured states with different grain sizes (10–200 nm) [16,17,18] or “amorphous nanoclusters” [20]. In addition, it was shown that SPD of the initially amorphous or initially crystalline alloy with 25 at.% Cu leads to the realization of several cycles of interrelated phase transformations of the “crystal-amorphous state” type [11].

In this work, we studied the effect of SPD under high pressure on the structural properties of the rapidly quenched TiNi–TiCu alloys with copper content exceeding the solubility limit of copper in TiNi (about 30 at.%). It was earlier established that an increase in the copper content substantially affects the structure formation in the alloys upon isothermal crystallization from amorphous state [11]. The single-phase B2 structure formed in the alloys with a Cu content below 34 at.% upon cooling undergoes transformation into the B19 martensitic phase. while the two-phase (B2 + B11) structure formed in the alloys with a Cu content of 34 at.% and above, in which the B11 (TiCu) phase inhibits the B2↔B19 martensitic transformation up to its complete suppression. In this regard, it was reasonable to expect significant differences in the characteristics of the alloys after SPD.

It was found that like the alloy with 25 at.% Cu, the amorphous alloys with a copper content of 30–40 at.% Cu upon HPT undergo nanocrystallization, which was detected using the XRD method. The SEM examination of the cross-sectional microstructure of the ribbons showed that HPT leads to the formation of structure elements with submicro- or nanoscale inclusions, presumably due to the partial crystallization of the amorphous phase. The fact that their number noticeably decreases with increasing copper content in the alloys can be associated with increasing degree of amorphization.

The study of the alloys by DSC revealed a difference in the character of crystallization after HPT of the alloys with high copper contents and the alloy with 25 at.% Cu. In the latter alloy, an increase in the degree of deformation broadens the crystallization peak and shifts it to lower temperatures, whereas the DSC curves of the alloys with 30–40 at.% Cu exhibit an additional isolated low-temperature peak indicating that regions are formed in which low-temperature crystallization is possible. A detailed examination of the structure formed in this case is expected in further studies.

As was established by the DSC studies, HPT of the alloys with a high copper content caused a decrease in the thermal effect of crystallization upon heating relative to that exhibited by the initial amorphous state after melt quenching. This suggests the presence of a crystalline phase in amorphous alloys after HPT [21]. Such changes cannot apparently be attributed to the elastic stresses stored upon HPT because they disappear at earlier heating stages in the process of structure relaxation [22].

The obtained results convincingly demonstrate the effect of copper content in the alloys of the TiNi–TiCu system on the structure formation upon torsion under high quasi-hydrostatic pressure. However, further research is necessary for a more detailed explanation of the combined effect of melt quenching, HPT, and crystallization parameters.

## Figures and Tables

**Table 1:** Crystallization enthalpy and peak temperatures of the TiNiCu alloys in the initial amorphous state and after HPT.

n	ΔH, J/g			T_p_, °C			T′_p_, °C			T″_p_, °C
	30Cu	34Cu	38Cu	30Cu	34Cu	38Cu	30Cu	34Cu	38Cu	38Cu
0	32.5	33.6	121.2	446.6	441.5	445.8	–	–	427.1	–
1	25.5	32.1	110.6	446.8	442.3	447.4	438.5	445.3	426.7	–
3	20.8	31.2	87.3	446.9	442.6	447.6	438.4	446.2	426.4	394.0
5	19.0	29.2	62.8	446.7	442.7	448.6	438.6	446.3	426.6	395.4
